# Refractory Nonvariceal Upper Gastrointestinal Bleeding Requiring Surgical Intervention

**DOI:** 10.7759/cureus.4135

**Published:** 2019-02-26

**Authors:** Edmund Hsu, Singwu D Law

**Affiliations:** 1 Emergency Medicine, Mount Sinai St. Lukes - Roosevelt Hospital Center, New York, USA; 2 Pulmonology, Mount Sinai St. Lukes and West Hospital Center, New York, USA

**Keywords:** massive upper gi bleed, therapeutic endoscopy, angiographic embolization, critical care medicine, emergency medicine, peptic ulcer disease, blood transfusion, massive tranfusion protocol

## Abstract

Nonvariceal upper gastrointestinal (GI) bleeds are a common emergency. Mortality in patients with an upper GI bleed has been reported to be as high as 30% for those who bleed inpatient. Definitive management after resuscitation can be done with endoscopy, transcatheter arterial embolization (TAE), and/or surgery. A 55-year-old female with multiple comorbidities presented with a refractory second episode of an acute nonvariceal upper GI bleed that required an interdisciplinary approach with the following interventions: endoscopy, embolization, and ultimately surgery. In this case report, the discussion is about the management algorithm of nonvariceal upper GI bleeds as well as the literature on prophylactic embolization and GI rebleeding. This unusual case presented with continued bleeding despite embolization, which led to the emergent rescue surgery that was necessary for this patient. Important take-home points are that patients with therapeutic hemostasis of upper GI bleeds may have rebleeding, a second attempt at therapeutic endoscopy after rebleeding may be limited due to a brisk bleed, the literature about prophylactic embolization is controversial, and one should involve both interventional radiology and surgery early on to assess a patient’s clinical picture for further definitive interventions from both specialties.

## Introduction

An upper gastrointestinal (GI) bleed is defined as any GI bleeding that occurs above the ligament of Treitz, and can be categorized into two types: variceal upper GI bleeds and nonvariceal upper GI bleeds. A variceal bleed is caused by dilated submucosal veins usually associated with increased hepatic portal pressure. A nonvariceal upper gastrointestinal bleed (NVUGIB) is most often caused by peptic ulcers that are associated with *Helicobacter pylori* infections and nonsteroidal anti-inflammatory drugs (NSAIDs) [1]. Upper GI bleeds are considered an emergency; mortality in patients with an upper GI bleed has been reported to be as high as 30% for those who bleed inpatient [2].

Current management of an acute NVUGIB begins with medical resuscitation and stabilization, which is followed by procedural intervention with endoscopy. In the past, if the first attempt at endoscopic hemostasis failed to control the peptic ulcer bleeding, then surgical intervention was used to induce hemostasis. In certain cases, early surgical intervention without re-endoscopy has been considered for patients with recurrent massive upper GI hemorrhage following initial endoscopic treatment [3].

Nowadays, alternative procedures to surgical intervention are more conservative. Angiography for visualization and transcatheter arterial embolization (TAE), introduced by Rosch et al. in 1972, as an alternative to surgery for upper GI bleeding, has been used as a diagnostic and therapeutic tool that is usually reserved for patients who are at high risk for surgery [4]. Newer studies have found that TAE is a safe treatment method for acute NVUGIB and a possible alternative procedure for high risk patients for surgery. However, the limitations of TAE are that embolization services are not readily available in every hospital and that there are risks, such as necrosis of the affected organ. Some studies advise that TAE be restricted to a subgroup of patients not primarily eligible for surgery once endoscopy has failed [4-5]. In this case, we will be reviewing the educational and clinical challenge of managing a refractory acute NVUGIB that required an interdisciplinary approach with interventions by endoscopy, TAE, and ultimately surgery.

## Case presentation

A 55-year-old morbidly obese female with insulin-dependent diabetes mellitus type 2 (IDDM2), hypertension (HTN), and hyperlipidemia (HLD) was admitted to the medical intensive care unit (MICU) for septic shock with a complicated hospital course, including an upper GI bleed due to a large ulcer on the anterior wall of the duodenal bulb with a pulsating vessel. Esophagogastroduodenoscopy (EGD) was performed and two clips were deployed on the bleeding vessel. Interventional radiology (IR) performed elective prophylactic arterial embolization and placed five coils in the gastroduodenal artery (GDA) with post-embolization contrast administration imaging which demonstrated lack of flow in the GDA.

**Figure 1:**
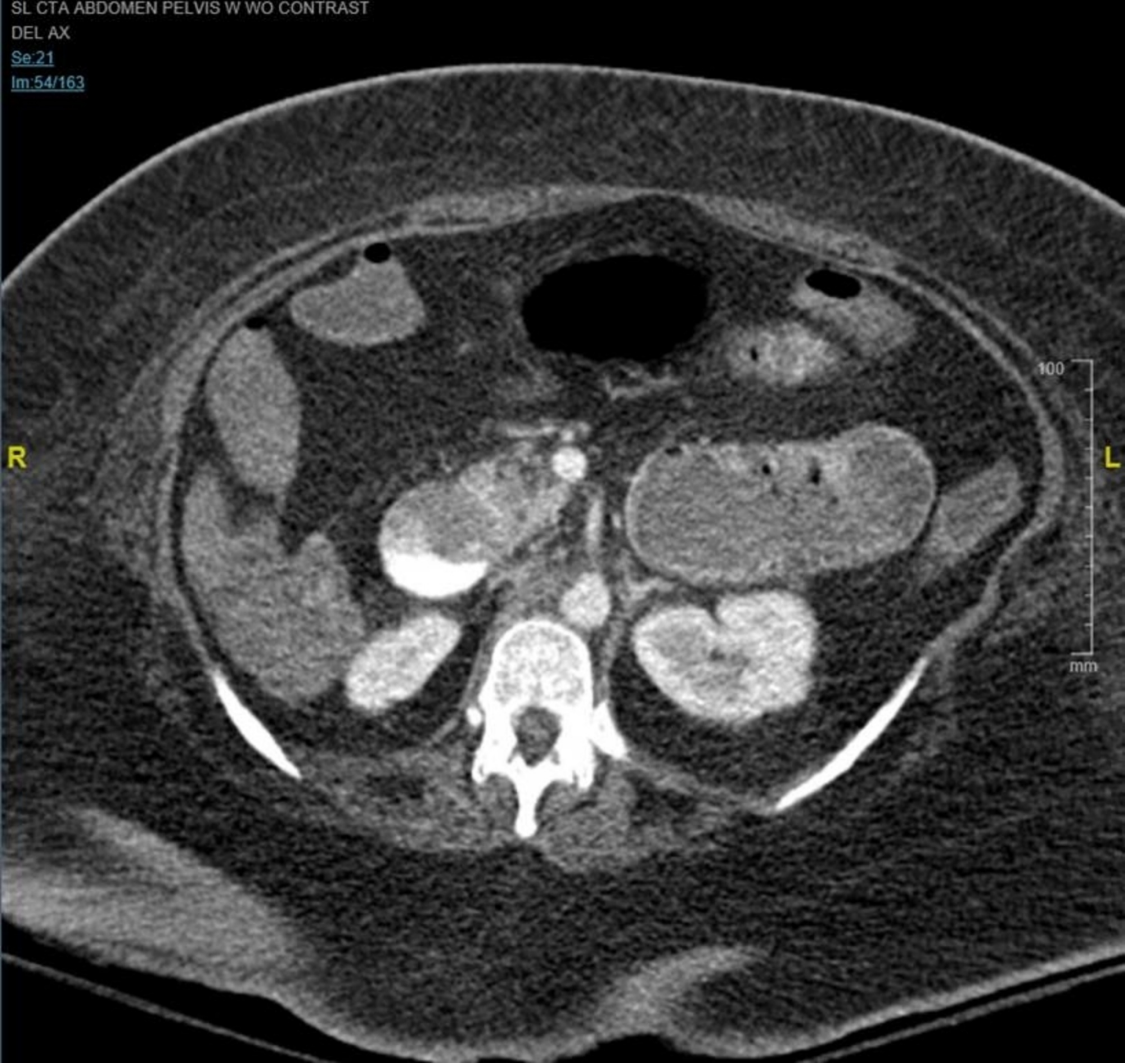
CT angiography abdomen and pelvis with contrast. Contrast extravasation within the duodenum compatible with gastrointestinal bleed.

The patient’s clinical course improved over the next 11 days and she was extubated with her blood pressure (BP) at 97/57. That evening, the patient was found with a BP at 50s/30s, worsening mental status, and over 1 L of melena on physical exam. GI was consulted stat for EGD, IR and surgery consults were called, massive transfusion protocol (MTP) was initiated, intravenous (IV) access was obtained, proton pump inhibitor (PPI) bolus was given, empiric antibiotics (abx), blood work was drawn, fluids and levophed was given, and anesthesia reintubated the patient. An arterial (A) line that was then placed measured systolic BP at 60s after five units of packed red blood cells (pRBC) and fresh frozen plasma (FFP). The patient was placed on vasopressin. The patient continued to have active melena with new bright red blood per rectum and hematemesis. She began second MTP and an EGD was attempted at bedside and aborted with the following findings: large amounts of clotted blood in the lower third of the esophagus and large amounts of bright red blood and clots in the entire stomach impairing visualization. The patient was started on third MTP and the computed tomography angiogram (CTA), as seen in Figure 1 below, showed active extravasation within the duodenum likely arising from the superior pancreaticoduodenal artery. The patient was taken to the IR suite, was started on the fourth MTP, was responsive to the MTP and pressors, and maintained a BP of 90s/70s. The results of IR intervention were as following: celiac angiography showed some coils packed in GDA, superior mesenteric artery (SMA) angiography showed active extravasation of contrast from the superior pancreaticoduodenal artery, successful glue embolization of the superiorpancreaticoduodenal artery, completion angiography of the celiac axis, and SMA showed no active extravasation. The patient returned from IR with a stable BP of 90s/70s. Over the next 20 minutes, over 1 L of bright red blood was collected from the oral gastric tube (OGT) suction without new melanotic stool and the BP started to decrease. The MTP number five was started and the patient went with surgery to the OR.

In the OR, surgery found and performed the following: in the peritoneal cavity, a distended stomach with blood was noted along with blood within the small intestine. A longitudinal duodenotomy was performed and bright red blood as well as clots was visualized. A 2-cm ulcer was noted in the posterior wall of the first portion of the duodenum. Within the ulcer, oozing of blood was noted. The previously placed endoscopic clip was noted. Also noted was what appeared to be coils, probably from the IR procedure. Two stay sutures were then placed at 12 o'clock and 6 o'clock positions using 3-0 silk suture material. Following this, exposure of the ulcer was obtained by suctioning the blood out. A 3-0 silk suture was then placed just superior to the ulcer in the posterior wall of the duodenum. This was then ligated. A similar suture was placed at 12 o'clock in the posterior wall of the duodenum just along the inferior edge of the ulcer. This was then tied down. A silk suture, using 2-0 silk, was then placed to ligate the transverse branch. Following this, hemostasis was obtained. The area was then irrigated and observed to make sure that there was no further bleeding. In the OR, the patient received another three units of pRBC, two units of FFP, and one unit of platelets. At this time the patient had a received a total of 28 units of pRBC, 27 units of FFP, and six units of platelets. The patient was then transferred to the surgical intensive care unit (SICU) and remained on the service for 10 more days with stable hemoglobin not requiring additional transfusion. During the SICU course, the patient had few episodes with melena, no episodes of bright red blood per rectum (BRBPR), and one episode of hematemesis. After 10 days the patient was transferred to the medicine floor team and is currently stable and clinically improving.

## Discussion

In summary, this case describes an actively hemorrhaging patient rebleeding status post 11 days from an endoscopic clipping and prophylactic coil embolization for a GDA NVUGIB that was managed with a re-trial of endoscopy, failed TAE, and ultimately rescue surgery.

Some 80% of acute upper GI tract bleeding will stop spontaneously. About 80% of those that have persistent bleeding achieve hemostasis with endoscopy, while the remaining percentage requires embolization or surgical intervention. It is estimated that bleeding can persist after successful endoscopic treatment with a rebleeding prevention rate of 80% [6]. Surgery is performed in fewer than 5% of patients with upper GI tract hemorrhage, but can be associated with operative mortality rates of up to 30% in patients with severe comorbidity [7]. The high mortality risk of surgery has been the main reason for the paradigm shift from surgical intervention after initial endoscopy to possible trial of arterial embolization. In the case of rebleeding in the past, emergency surgery was classically considered as the treatment of choice; however, it is now recommended to treat a rebleeding with a second therapeutic endoscopy procedure [8-9].

However, an article published in 2001 by Schoenberg stated that surgical intervention should be considered over a second therapeutic endoscopy in certain cases. In that article, Schoenberg stated that a subgroup of elderly patients who are suffering from hypotension due to rebleeding having large ulcers, and afflicted with several other illnesses, should undergo surgery immediately because endoscopic intervention often fails and that these patients would deteriorate quickly [10]. In the case of this patient, surgery was consulted about whether or not to pursue immediate surgical intervention; this patient was a poor candidate for surgery because she was suffering from other ongoing illnesses as well as hypotension due to rebleeding. Even though the patient was a poor surgical candidate due to comorbidities and body habitus, the patient did benefit from the first endoscopy during the first episode of GI hemorrhage, which pushed the discussion towards a final decision to plan another endoscopy.

Another avenue to consider is TAE before endoscopy which has mixed conclusions in the literature. One argument is that performance of angiography and TAE before endoscopy leads to an unacceptably high frequency of unnecessary angiography and the benefits are that endoscopic diagnosis and therapy can render angiography unnecessary. The flip side is that Loffroy et al. found that longer time to angiography is a predictor of early rebleeding after TAE. They concluded that every effort should be made to perform angiography with embolization early after bleeding onset [11].

This recommendation was taken into consideration for the case of rebleeding and a second endoscopy was attempted, but was limited due to poor visualization with active hemorrhaging. This pushed us to proceed with more definitive interventions and the patient underwent TAE with glue embolization.

Current literature states that TAE is preferred to surgery in elderly and other high-risk patients because it is not as invasive as surgery and has fewer complications. The retrospective analysis done by Ripoll et al. found that there was no difference shown between embolotherapy and surgery despite older age and greater prevalence of heart disease in patients receiving the embolotherapy [12].

While embolization has become an important step in the management of NVUGIB, this patient 11 days prior had received prophylactic embolization and still had an episode of rebleeding. The possibility of rebleeding after prophylactic embolization is not new. Širvinskas et al. found in their study a mean rebleeding rate of 27.8%. Significant associations were found between rebleeding and prophylactic embolization (OR = 10.53; p = 0.04), mortality and prophylactic embolization (OR = 10.53; p = 0.04), and units of pRBC and prophylactic embolization (OR = 1.25; p < 0.01) [5]. Other studies have looked at clinical factors that may help to predict risk of rebleeding. Loffroy et al. reported additional clinical factors associated with rebleeding such as cirrhosis, previous surgery, and massive blood loss [13]. Previous studies have shown that predictors of rebleeding were: coagulopathy, longer time to angiography, massive transfusion, previous surgery, bleeding secondary to trauma, cancer bleeding, use of coils as the only embolic agent, or multiorgan failure. On the other hand, several studies reported no difference of outcomes between patients who underwent prophylactic embolization and those who had embolization only after the site of bleeding was located during angiography; the topic of prophylactic embolization remains controversial [14].

In this case, the patient had prophylactic embolization the first time from the GDA. Dempsey et al. reported recurrent bleeding (30%) after prophylactic GDA embolization [15]. The patient then had a therapeutic embolization for the second time. Širvinskas et al. found an association between early rebleeding and therapeutic or prophylactic embolization. A possible mechanism for the repeated bleeding could be explained by the abundant collateral circulation of the duodenum and/or inaccurate artery selection when performing a prophylactic embolization [5]. Ultimately, the CTA and the angiography showed that the patient was bleeding from the superior pancreatoduodenal artery when the initial bleed was from the GDA.

## Conclusions

Even though many studies have compared and contrasted the results of GI endoscopy followed by embolization or surgery, this interesting case is one of the few that highlights the management of an acute NVUGIB with endoscopy, followed by embolization, and ultimately surgery. Many studies compare GI bleeds that are refractory to endoscopy than embolization or surgery. The studies also compare when to perform embolization versus surgery. However, this rare case presented with continued bleeding despite embolization, which led to the emergent rescue surgery that was necessary in this patient. As a poor surgical candidate due to her underlying health illnesses, this patient had therapeutic endoscopy and prophylactic embolization prior to her GI rebleed. Important take-home points are that patients with therapeutic hemostasis of upper GI bleeds may have rebleeding, a second attempt at therapeutic endoscopy after rebleeding may be limited due to a brisk bleed, the literature about prophylactic embolization is controversial, and one should involve both IR and surgery early on to assess the patient’s clinical picture for further definitive interventions from both specialties.
